# Regulatory Dynamics of Plant Hormones and Transcription Factors under Salt Stress

**DOI:** 10.3390/biology13090673

**Published:** 2024-08-29

**Authors:** Muhammad Aizaz, Lubna, Rahmatullah Jan, Sajjad Asaf, Saqib Bilal, Kyung-Min Kim, Ahmed AL-Harrasi

**Affiliations:** 1 Natural and Medical Science Research Center, University of Nizwa, Nizwa 616, Oman; 2 Department of Applied Biosciences, Kyungpook National University, Daegu 41566, Republic of Korea; rehmatbot@yahoo.com (R.J.); kkm@knu.ac.kr (K.-M.K.)

**Keywords:** salinity, plant response, hormones, ABA, JA, signaling

## Abstract

**Simple Summary:**

The current review article focused on the complex interaction between plant hormones and transcription factors in the response to salt stress, a pressing global issue that has a considerable effect on agricultural productivity. The study unveils the effect of salt stress on the ion equilibrium and triggers a cascade of molecular reactions that have a response to plant growth and development. Transcription factors are essential in regulating gene expression under salt stress. The transcription factors function in collaboration with hormones to regulate environmental stress responses. The study sheds light on the underlying regulatory networks, thus providing crucial knowledge for cultivating salt-tolerant crops through selective breeding and genetic engineering. This review significantly contributes to the establishment of sustainable agricultural techniques, which are essential in addressing the increasing problem of soil salinization and securing global food security.

**Abstract:**

The negative impacts of soil salinization on ion homeostasis provide a significant global barrier to agricultural production and development. Plant physiology and biochemistry are severely affected by primary and secondary NaCl stress impacts, which damage cellular integrity, impair water uptake, and trigger physiological drought. Determining how transcriptional factors (TFs) and hormone networks are regulated in plants in response to salt stress is necessary for developing crops that tolerate salt. This study investigates the complex mechanisms of several significant TF families that influence plant responses to salt stress, involving AP2/ERF, bZIP, NAC, MYB, and WRKY. It demonstrates how these transcription factors (TFs) help plants respond to the detrimental effects of salinity by modulating gene expression through mechanisms including hormone signaling, osmotic stress pathway activation, and ion homeostasis. Additionally, it explores the hormonal imbalances triggered by salt stress, which entail complex interactions among phytohormones like jasmonic acid (JA), salicylic acid (SA), and abscisic acid (ABA) within the hormonal regulatory networks. This review highlights the regulatory role of key transcription factors in salt-stress response, and their interaction with plant hormones is crucial for developing genome-edited crops that can enhance agricultural sustainability and address global food security challenges.

## 1. Introduction

### 1.1. Overview of Salt Stress in Plants

Plant breeding is severely hampered by soil salinization, which is also harmful to agricultural productivity, yield, and crop growth worldwide [1]. The main challenge in producing plants in highly salty soils is the disturbance of the water cycle balance. The two terms “primary salt harm” and “secondary salt harm” indicate the negative impacts that salt-stress effects on plants. Salt damage occurs by the direct influence of salt ions, which may lead the cell membrane to show significant harm. Secondary salt damage is the term for the indirect impacts of salt ions that produce osmotic stress, which inhibits the plant from absorbing water [2]. The concentration of the soil solution is induced as the concentration of salt in the soil rises. Osmotic stress induction can significantly reduce a plant’s capability to absorb water [3]. The plant observes that it is difficult to consume water from the ground when the concentration of salt in the soil increases too much. The soil solution has a greater osmotic pressure than that of the plant cells. This condition may lead to plant roots being dehydrated, producing a situation similar to a physiological drought and, in extreme situations, possibly resulting in the plant’s death [4]. Due to the alteration of the lipid bilayer organization and the structure of membrane proteins, salt stress negatively impacts cell membranes, resulting in increased membrane lipid permeability, leading to lipid peroxidation [5,6]. Consequently, these changes affect the natural physiological functions of the membrane [7]. Under salt stress, plants lose water from cells, leading to alterations in cell turgor and osmotic potential. The destruction of both the cell membrane and plasma membrane results in a reduction or loss of selective permeability in the cell membrane. Consequently, this causes the leakage of beneficial ions like Ca^2+^ and K^2+^ from the cell [8]. Simultaneously, toxic ions such as Na^+^ and Cl^−^ accumulate within the cell, causing an ion imbalance and further compromising cellular integrity. Cell membrane, organelle membrane, organelle structure, chlorophyll content, and photosynthetic rate lead to decreased and enhanced ribonuclease activities [9]. This leads to stress damage, weakening the plant’s resistance [10,11].

### 1.2. Importance of Understanding Plant Responses to Salt Stress

**Figure 1:**
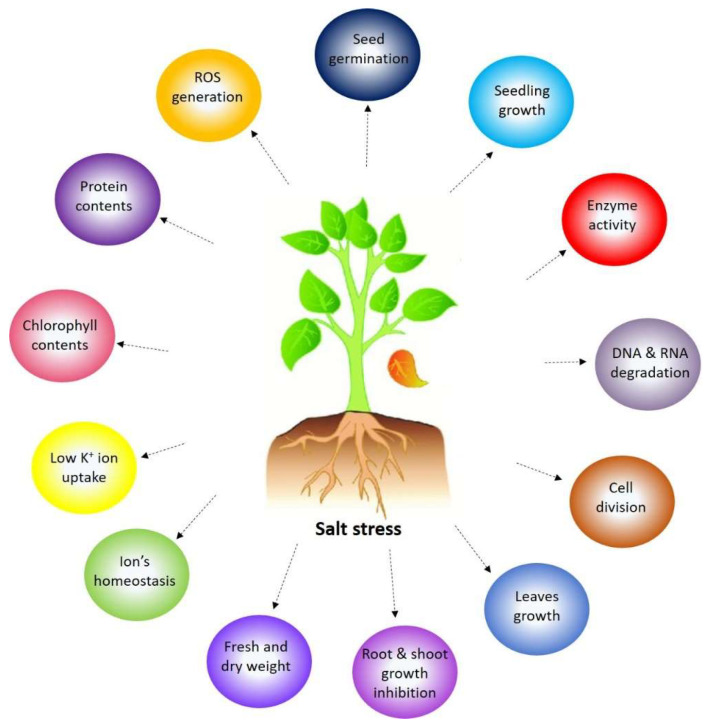
The morphological, physiological, and biochemical response of a plant under salinity stress.

During salt stress, plants show different responses efficiently expressing physiological attributes that adapt to or alleviate the impact of salt stress [12,13]. Figure 1 shows the effect of salt stress on plant growth and development. At low salinity concentrations, the development of the root is generally lower influenced than shoot growth, resulting in an increased root-to-shoot ratio [14]. In contrast, higher salt concentration decreases the root growth [15]. With increased salinity stress, the plant experiences a significant decrease in the accumulation of dry biomass due to a reduction in shoot and root growth [16]. It has been previously reported that inhibition of growth may occur due to reduced rates of new cell formation [17]. Salinity-induced changes in the cell wall structure may have contributed to the cell wall’s increased stiffness and decreased dry weight accumulation. Osmotic stress, which affects salt stress in the root zone, impairs cell ion homeostasis by causing both an increase in the accumulation of Na^+^ and Cl^−^ and an inhibition of the consumption of vital nutrients like K^+^ [18]. Increased uptake of Na^+^ competes with the absorption of other essential nutrient ions, particularly K^+^, resulting in a deficiency of potassium, which causes a lower K^+^/Na^+^ ratio in plants under salt stress [18]. Plants under salt stress exhibit notable alterations in their physiological and biochemical characteristics, including reduced levels of chlorophyll content in their leaves, a decline in protein synthesis, an increase in reactive oxygen species (ROS) accumulation, an increased accumulation of compatible solutes like proline, and modifications in antioxidant enzyme activities. Therefore, the total changes in plant growth and productivity are caused by the combination of all the morphological, physiological, and biochemical modifications in plants exposed to salt stress.

### 1.3. Significance of Transcriptional Factors and Hormonal Networks in Salt-Stress Response

It has recently been discovered that NaCl stress has a significant impact on the hormonal balance and the expression of specific transcription factors. Phytohormones, including abscisic acid (ABA), salicylic acid (SA), and jasmonic acid (JA), were shown to increase, whereas the levels of gibberellic acid (GA) decreased. Additionally, there was an initial increase and then a decrease in trans-zeatin (tZ) and indoleacetic acid (IAA) [19]. RNA-seq was used to completely investigate the expression patterns of genes associated with hormone production and signal transduction. As a result, a coextrusion network involving hormones and a genome-wide co-expression network were constructed using weighted gene co-expression network analysis (WGCNA). The study suggested a transcriptional regulatory model including six different hormone types and identified 20 hormone-related candidate genes connected with salt stress. This provides a significant understanding of the molecular mechanisms and insights into tomatoes’ abilities to tolerate salt [19]. Modulating gene expression in response to salt stress is mostly dependent on transcription factors. In combination with hormones, biotic and abiotic factors, symbiotic relationships, cell differentiation, and stress-signaling pathways, they intricately control gene expression. Additionally, by regulating the expression of downstream target genes, transcription factors (TFs) contribute significantly to the development of plant salt tolerance. For instance, when ABA binds to PYL, it reduces negative regulation, activating AREB/ABF transcription factors, which are bZIP proteins that bind to ABA response elements in the promoter regions of target genes [20]. It has been studied that endogenous ABA levels increase rapidly after oxidative stress, acting as a signaling molecule that regulates stress-responsive genes, such as DREB proteins and bZIP, to enhance plant productivity through drought stress tolerance mechanisms [21]. The crosstalk between transcription factors and hormones under saline conditions involves the coordination of stress-responsive transcription factors, such as those from the AP2/ERF, MYB, and bZIP families, with plant hormones like ABA and ethylene. This interaction regulates the expression of antioxidant genes, helping to maintain reactive oxygen species (ROS) homeostasis and enhancing plant tolerance to salt stress. Furthermore, primary osmotic stress, ion toxicity, secondary oxidative stress, and nutritional stress are all addressed by this control, which can help reduce the adverse effects that salt stress causes to plants [22,23]. Plant responses to salt stress are mostly mediated by the hormonal networks, particularly those involving ethylene. The formation of ethylene during salt stress suggests it is important for the salt response. In addition to regulating proteins at the post-translational level, salt stress stimulates specific genes at the transcriptional level. This complicated mechanism controls the complex interaction between gene expression and hormone signaling in salt-stressed environments [24]. An investigation of the association between hormones and transcription factors under salt stress was conducted on tomato plants. A complex transcriptional regulatory network has been revealed by a detailed examination of the expression patterns of transcription factors (TFs) and candidate genes connected with hormones under salt stress. The complex interactions between gene expression and signaling pathways play a significant part in the ability of plants to respond to salt stress [19].

## 2. Transcriptional Factors in Salt-Stress Regulation

### 2.1. Role of TFs in Gene Expression Regulation

The regulation of plant responses to the growing worldwide problem of soil salinization is mainly reliant on transcription factors (TFs). A comprehensive study into their function and the several families of transcription factors activated in the regulation of salt stress [25]. There are significantly more connections between the regulation of salinity stress and transcription factor families such as AP2/ERF, bZIP, NAC, MYB, and WRKY [26]. Two more important TF families that are linked to the ability to deal with abiotic stress like salt stress are ABF and DREB [27]. Specific DNA sequences are linked by transcription factors (TFs) that regulate the rate at which genetic information is transcriptionally transformed from DNA to messenger RNA. Thus, the regulatory process facilitates the expression of genes that are essential for salt-stress tolerance and adaptation in plants [28]. Transcription factors (TFs) have a major impact on signal-transduction networks that respond to salt stress in plants. They are a part of the mechanisms that alert the plant’s genetic machinery to the presence of salt stress, which causes stress-responsive genes to express themselves [23]. TFs contribute to salt tolerance in crops by signaling and activating genetic responses, which are crucial for ion homeostasis, osmotic stress pathway activation, and plant hormone signaling, enabling plant survival under salt stress [28]. To illustrate the specificity and critical role certain TF families play in responding to salt stress, bZIP Transcription Factors are specifically linked to the regulation of plant salt stress [29]. Soil salinity has a major impact on crop development and yield. It is imperative to understand and manipulate TFs to advance plant breeding and agricultural advancements [29].

### 2.2. Key Transcriptional Factors Involved in Salt-Stress Response

TFs are proteins that bind to DNA regulatory sequences, found upstream in the 5′ region of target genes, and control gene transcription. Consequently, TFs play a crucial role in activating and deactivating specific genes by binding to other DNA sequences and regulating gene transcription and protein synthesis, leading to modifications in cellular function within plant tissues [27]. TFs are highly conserved in the plant genome, altering gene expression and endowing plants with resilience to salinity. Some TFs are generally found in all cells of a plant.

There are various cell types and stages of development for which a few transcription factors are specialized. These transcription factors are essential for controlling the expression of genes, which affects development. It is crucial for understanding the transcription mechanism because it regulates changes in gene transcription. In all eukaryotes, the enzyme in charge of transcription is called RNA Polymerase II, or RNAP II. However, RNAP II cannot function on its initiative. Trans-acting factors, which are transcription factors, and cis-regulatory regions, which are certain DNA sequences within or closer to a gene, control its activity. Transcription factors are necessary for RNAP II to be able to transcribe DNA throughout this process. Particular DNA sequences known as “cis-acting elements” are essential for regulating transcription [30]. The TF families bZIP, MYB, NAC, and WRKY are associated with stress tolerance [31].

### 2.3. Role of Stress-Responsive Transcription Factors in Enhancing Abiotic Stress Tolerance in Plants

Stress-responsive transcription factors are significant for the development of stress tolerance as well as abiotic stress responses [32]. Therefore, targeting stress-responsive transcription factors is essential for developing crops that are more resilient to abiotic stress, ensuring enhanced tolerance [33]. Genetic engineering is recognized as an alternative strategy for enhancing stress tolerance in crops by significantly changing their agronomic characteristics. Abiotic stress response has been identified in numerous genes, including functional proteins and proteins involved in signal-transduction pathways [34]. Also known as trans-acting factors, they are essential components in the signal-transduction pathways for abiotic stress. Transcription factors bind to different cis-elements and regulate the expression of a family of related genes, which is essential to the mechanism of plant resistance. The transcriptional regulation domain, nuclear localization signal (NLS), oligomerization site, and DNA-binding domain are the four fundamental parts of transcription factors. The combined action of these structural domains determines the time, space, and mode of action of the regulatory functions of transcription factors [35]. Transcription factors can be divided into several categories according to the characteristics of DNA-binding regions. In these responses, WRKY, bZIP, MYB, and NAC. Transcription factors play key roles in various physiological processes in various plants shown in Table 1, Table 2, Table 3 and Table 4. Under salt stress, the dynamic changes in hormones had a significant effect on the normal growth and development of plants [24]. Salt stress may be reduced by the buildup of Abscisic acid, which can regulate stomatal closure, ion homeostasis, gene expression, and metabolic changes in response to salt stress [36]. When plants were exposed to salt stress, the accumulated Indole acetic acid and Cytokinin in root tips could confer augmented resistance [24]. Previous research has shown that under salt stress, the expression of the IAA-related genes SAUR32, SAUR36, and ARF5, as well as the gene related to cell division, IPT5, could be significantly induced in the roots of apple rootstocks. These genes could improve salt tolerance by increasing the IAA and CK content in the apple rootstocks [37]. Jasmonic acid activation of antioxidant enzymes can delay flowering, reduce plant growth and root elongation, and increase plant viability under salt stress [38]. Additionally, Salicylic acid and Gibberellins were also crucial in salt stress [39].

## 3. Major TF Families 

In this study, we focused on four TF families implicated in abiotic stress tolerance, and the significance of these transcription factors in salt-stress signaling is described in Table 5.

## 4. Mechanisms of TF Activation during Salt-Stress 

Complex networks characterize plant abiotic stress signal-transduction processes. One environmental stimulus can cause several secondary stress signals in plant cells, and each of these secondary stress signals might be transmitted through a various signaling pathway [127]. This shows a challenge for developing a thorough understanding of TF functions in plant signaling pathways. Nevertheless, these pathways may eventually regulate the same target genes or exhibit crosstalk at certain nodes during the signal-transduction cascades [128]. Abiotic stress-signaling pathways may exhibit crosstalk toward single or multiple stressors. To address this, molecular analyses of stress-induced genes using RNA-seq, oligo arrays, or full-length cDNA microarrays are several ways to investigate [129]. Such strategies have demonstrated that plants express crosstalk variously in response to drought and salt stress compared to their response to drought and cold stress. Most drought-inducible genes were also triggered by high salinity and ABA treatments, according to the recent finding that 10% of drought-inducible genes could be activated by cold [130]. Abiotic stress responses in plants can be regulated by TFs from different families, either independently of ABA (*CaNAC05* and *CaNAC41*, for example) [131] or dependently on this hormone (such as *AtAREB1* and *GhWRKY17*) [132]. Alternatively, they can function in both ABA-dependent and ABA-independent pathways (such as *TaMYB19*, *MbDREB1*, and *DREB2A*) [133]. Target gene promoters contain significant cis-elements that can interact indirectly with TFs to produce crosstalk. The abiotic-stress-responsive RD29A promoter, for example, has both DRE/CRT and ABRE cis-elements that can be bound by TFs that bind DRE/CRT (*AtDREB1* and *AtDREB2*) and ABRE (*AtAREB1* and *AtAREB2*), respectively. Transactivation studies in Arabidopsis have demonstrated that these TFs work together to cooperatively regulate the target gene’s expression [134]. This investigation further shows that the degree of RD29A transcriptional activity depends on a combination of DREB/AREB regulators simultaneously to the promoter’s cis-elements. Different studies on TF binding to the *Arabidopsis RD29A* promoter indicated that this promoter also contains the binding site (named NACRS) for ANAC096, and TF was able to bind with *AtAREB1* and *AtAREB2* in proximity and NACRS and ABRE regions [135]. Furthermore, studies performed by Oh et al. suggested that the target gene might be more probable to be highly regulated by AREBs or DREBs if there were more DRE or ABRE sequences in the promoter region, which contains both cis-elements [136]. Various transcription factors have been assigned as multi-functional regulatory proteins that engage in both biotic and abiotic stress pathways. This has been demonstrated by the NACs from Arabidopsis [ATAF1 (Arabidopsis transcription activation factor 1) and ATAF2] and rice (*OsNAC6*) [137], and by MYBs from wheat (*TaPIMP1*) and rice (*OsMYB4*) [138], and WRKY TFs from rice (*OsWRKY45*) and grapevine (*VvWRKY11*) [139].

## 5. Hormonal Networks in Salt-Stress Response

### 5.1. Overview of Plant Hormones Involved in Salt-Stress Response

Plant hormones play a crucial role in how plants respond to salt stress, which is a significant environmental stressor that limits growth and productivity. There are nine well-characterized plant hormones, and among these, abscisic acid (ABA), ethylene, salicylic acid (SA), and jasmonic acid (JA) are specifically recognized for their roles in stress response. Meanwhile, others like auxin, gibberellin (GA), cytokinins (CKs), brassinosteroids (BRs), and strigolactones (SLs) are primarily associated with growth promotion [24]. Hormonal regulation is part of a sophisticated network of biological mechanisms that plants employ to adapt to salt stress, involving osmoregulation, redox, and ionic homeostasis, as well as adjustments in growth through hormone or light signaling-mediated pathways [140]. Interaction between these hormones contributes to a complex regulatory system that allows plants to sense, signal, and respond to the harsh conditions imposed by high salinity levels [141]. The balance and interaction between these hormones and their receptors play a critical role in determining the plant’s response to salt stress. For instance, the dominance of receptor signaling over ethylene might make the plant more susceptible to salt stress [142]. This crosstalk among hormones underlines the importance of both stress and growth hormones in plant adaptation to salt stress [24]. Specifically, one significant phytohormone that is crucial for responding to various stress signals is ABA. When ABA is applied to plants, it simulates the effects of stress. The expression patterns of stress genes and ABA treatment overlap because numerous abiotic stimuli lead to cell desiccation and osmotic imbalance. It suggests that to maintain cellular homeostasis, numerous stress signals and ABA might have similar parts in the signaling pathway that communicate with one another [143]. Abscisic acid (ABA) is essential for many physiological functions, such as reducing the germination of seeds and causing dormancy, controlling seed growth, encouraging stomatal closure, controlling embryo morphogenesis, facilitating the synthesis of lipids and storage proteins, accelerating leaf senescence, and promoting defense mechanisms against infections [144]. The main function of ABA seems to be the regulation of plant water balance and osmotic stress tolerance. Several ABA-deficient mutants namely *aba1*, *aba2*, and *aba3* have been noted in Arabidopsis [145].

### 5.2. Interplay between ABA, Salicylic Acid (SA), Jasmonic Acid (JA) and Other Hormones

A well-organized mechanism that involves the plant’s capacity to adapt to changing environmental conditions determines the way plants respond to abiotic stressors. A signal-transduction cascade’s crosstalk is the point at which several hormones interact with one another. Salicylic acid, JA, and ABA are essential phytohormones in stress signaling. For stomatal closure, ABA always triggers JA by a Ca^2+^ influx, which mediates the CDPK signaling cascade. Studies further demonstrated that PYL6 (*RCAR9*) and an ABA receptor with the corresponding transcription factor MYC2 influenced the expression of JAZ6 and JAZ8, indicating a possible association between ABA and JA in stress tolerance [146]. Cytokinin hormone is also involved in delayed senescence, cell growth, and leaf expansion. ABA and CK, however, have been found to be negatively correlated, which eventually results in stomatal closure and reduced water loss [147]. The CK receptor kinase, which significantly regulates ABA levels, has also been found to be responsible for a relationship between Arabidopsis histidine phosphotransferase proteins; however, stress tolerance is additionally observed in CK-deficient mutants [148]. On the other hand, by inhibiting ABA in response to dryness, ethylene has demonstrated a negative correlation between gas exchange and the development of leaves and roots [149]. Increasing ABA levels caused ABI5 to be triggered, which controls LEA genes that serve as an osmoprotectant for the seed under stress [150]. Additionally, ABA and GA showed an antagonistic interaction, and their balance regulated the dormancy and germination of seeds. The interplay between ABA and GA signaling under abiotic stress conditions is significantly influenced by DELLA proteins. The RINGH2 factor, which encodes XERICO and mediates ABA signaling, ABA accumulation, and ABI5 activity to disrupt GA levels, has been observed to be induced by RGL2, a protein related to the DELLA protein family [151]. Thus, by inactivating through the 26S proteasome pathway and increasing the GA level, RGL2 has been identified to be a crucial factor in breaking seed dormancy [152]. Because RGL2 regulated MFT for phosphatidylethanolamine binding protein, which showed an adverse relationship with ABI5 but a positive correlation with ABI3, MFT was additionally identified in ABA and GA signaling [153]. In phytohormone-mediated agricultural plant growth and development under environmental stress, ABA primarily functions via interacting with other related plant hormones.

### 5.3. Hormonal Crosstalk in Salt-Stress Adaptation

Physiological drought is caused by salt stress, which leads to degraded protein and photosynthesis [154]. Reactive oxygen species (ROS) formation, excessive amounts of sodium ions (Na^+^), and changes in intracellular Ca^2+^ levels are examples of signals that trigger the salt-stress response [155]. Ethylene is the primary hormone in salt-stress response [156]. The amounts of ET and its precursor ACC (1-aminocyclopropane-1-carboxylate) increase under NaCl stress [157]. Production of ET synthesis may induce salt sensitivity [154]. Five ethylene receptors (*ETR1*, *ERS1*, *ETR2*, *EIN4*, and *ERS2*), a protein kinase known as CTR1 (Constitutive Triple Response 1) functioning as a negative regulator, and an essential positive regulator known as EIN2 are all involved in the intricate process of ethylene signaling. Associated with several downstream ethylene response factors, this signaling route further activates main transcription factors like EIN3, EIL1 (Ethylene Insensitive Like 1), and EIL2. Osmotic stress, which is produced by several abiotic variables, including salinity, prevents ETR1 (Ethylene Response 1) expression [154]. Cotton showed upregulation of ethylene receptor genes (*ETR1*, *ETR2*, and *EIN4*), signaling genes (*CTR1*, *EIN3*, *ERF1*, and *ERF2*), and MAPK cascade genes (MEKK1-MKK2-MPK4/6) in response to both short- and long-term salt stress [158]. Ethylene and salt stress both affect the many Ethylene-Responsive Element Binding Factor (*ERF*) genes, particularly *ESE1–ESE3*. *EIN3* accumulation and transcriptional activity are enhanced, and *EBF1*/*EBF2* is degraded by salt stress [158]. When exposed to salt stress, 1-aminocyclopropane-1-carboxylic acid synthases (ACSs) are significantly expressed [159]. Salt stress tolerance in Arabidopsis seedlings increases with ACC pretreatment [160]. Salt causes the tobacco to induce ACS1 transcripts [161]. Four ACS genes were upregulated in both salt and non-acclimated plants under salt stress [162]. Stress-triggered (MAPK) cascades phosphorylate ACSs during the post-transcriptional regulation of synthases (ACSs), preventing the 26S proteasome from degrading ACSs detrimentally [156]. The effects of salt acclimatization are reduced by the decrease in MAPK6 function [162]. Stabilization of ACSs apparently needs MPK6 to maintain high ethylene levels. ACSs are also stabilized by CDPKs (Calcium-Dependent Protein Kinases) in tomatoes and ACC content and activity of 1-aminocyclopropane-1-carboxylic acid oxidase (ACO) is increased under salt stress in Cicer arietinum roots [156]. ABA modulates several genes that are sensitive to stress [163]. ABA and ET collaborate to mediate salt stress. Several genes involved in ABA production, including ZEP, AAO, and MCSU, are activated during exposure to salt via downstream signaling pathways and Ca^2+^ dependent phosphorylation processes [164]. It has been found that certain plants, including *Oryza sativa* [165] *Brassica* [166], and *Zea mays* [167], demonstrated significant ABA levels. Increased levels of ABA lead to stomatal closure and assist in the accumulation of proteins for osmotic modification. Salt-stress tolerance is enhanced by high accumulation of ABA due to ectopic expression of drought-responsive genes in rice, particularly *OsCam1–1* (Oryza Sativa Calmodulin1–1) and *OsDSM2* (Drought-Hypersensitive Mutant 2) [163]. Many MAPKs are upregulated in response to salt stress and ABA treatment, and plants that express MAPKs are more tolerant of salt stress [163]. Salt stress is influenced by ABA-regulated Ca^2+^-dependent kinases and *SnRks*, as well as phosphorylate ABA-related transcription factors, and affects gene expression [168]. Several regulatory sequences, including DRE/CRT, ABRE, MYC recognition sequence (MYCRS), and MYB recognition sequence (MYBRS), have been identified in the promoters of stress-responsive genes. ABA-dependent transcription factors (ABFs), MYCs, and MYBs directly bind to specific regions on the promoters of salt-stress-responsive genes and promote their activation. All LEA genes have ABRE motifs in their promoters, which bind ABF. Dihydroorotate Dehydrogenase1 is a drought-inducible gene that is essential to salt and drought stress responses, and it is regulated by ABFs and DREB2 [163]. The mutation in ACS7 reduces tolerance to heat, osmotic, and salt stress due to the crosstalk between ethylene (ET) and (ABA) during stress conditions [156]. There is limited information reported on the salt-stress mechanism via auxins [163]. Induced hypersensitivity to salt stress leads to increased auxin production caused by the *UCCA3* gene, which is involved in auxin biosynthesis [169]. Changes in the root architecture are caused by auxin accumulation and redistribution in response to salt stress [170]. In previous studies, we reported that auxin levels significantly decreased in tomato under salt stress [171]. Salt stress leads to decreasing growth, an indication of changes in both IAA biosynthesis levels and distribution, and it decreases CK production in wheat [172]. The Na^+^ transporter-encoding *HKT1-1* gene was expressed more often in mutants with lower CK levels [173].

## 6. Crosstalk between Transcriptional Factors and Hormonal Networks

### 6.1. Interaction between TFs and Hormone Signaling Pathways

**Figure 2:**
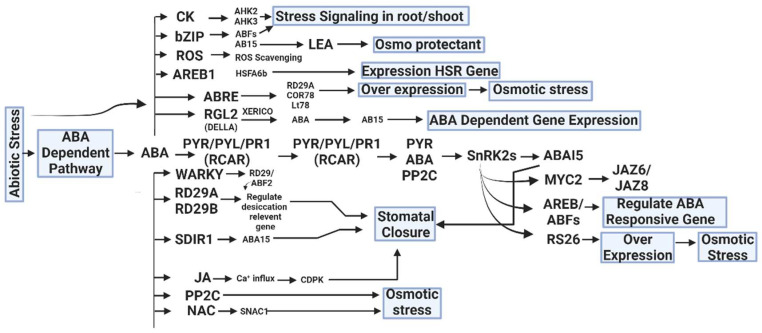
Hormonal crosstalk and abiotic stress tolerance are caused CK (Cytokinin), ROS (Reactive oxygen species), LEA (Late Embryogenesis Abundant), AREB1(Abscisic Acid Response Element Binding Protein 1), RGL2 (Repressor of GA LIKE 2), PYR (Pyrabactin Resistance Gene), RD29A (Response-to-Dehydration 29A), SDIR1 (Salt and Drought-Induced Ring Finger), JA (Jasmonic Acid), PP2C (Protein Phosphate 2C), JAZ (Jasmonate-ZIM domain), and SnRK2s (Sucrose Non-Fermenting 1-Related Protein Kinase 2s) by an ABA-dependent and independent pathway [150].

The complex interactions between hormone signaling pathways and gene regulation are largely dependent on transcription factors (TFs) (Figure 2). They act as key regulators of essential cellular processes, including differentiation, development, and the cellular response to external signals [174]. Hormone signals often first impact transcription factors, which then determine how biological pathways will develop, such as during somatic embryogenesis [175]. In plants, for instance, the cross-regulation between different hormone signaling pathways can influence the activity of TFs. Researchers have examined this cross-regulation, identifying network components that might be responsible for such interactions. There is a large-scale protein–protein interaction network that provides a comprehensive analysis of this cross-regulation in plant hormone signaling [176]. Furthermore, the activity of transcription factors in response to plant hormones has been extensively characterized, and dynamic transcriptional regulatory models for several hormones, including abscisic acid, brassinosteroid, ethylene, jasmonic acid, salicylic acid, and strigolactone/Karrikin, have been reconstructed to understand these relationships better [39]. A specific instance of TFs interacting with hormone signaling pathways can be seen in the Arabidopsis transcription factor LONG HYPOCOTYL 5 (HY5). This factor plays a significant role in photomorphogenic development by promoting the expression of genes that are negative regulators of auxin signaling, therefore linking hormone and light signaling pathways. Mutations in HY5 disrupt this balance, leading to altered hormone signaling and plant development phenotypes [177]. Moreover, in the context of stress responses, WRKY transcription factors in plants have been identified as key players in regulating hormone signal-transduction pathways. These TFs are integral to plant processes responding to both biotic and abiotic stress, suggesting their pivotal role in hormone-mediated stress-response mechanisms [178].

### 6.2. Feedback Mechanisms Regulating TFs and Hormonal Responses

The cross-regulation of hormone signaling pathways plays a major role in plant growth and development [179]. Plants utilize this mechanism to analyze several types of environmental and internal signals from their surroundings, absorb and interpret the data, and then initiate appropriate responses. This enables plants to respond to stressors, show plastic growth, and adapt to their local environment [180]. The growth defense trade-off is a well-known example of when plants, under pathogen attack, prioritize the allocation of resources to defense mechanisms [181]. Nevertheless, this trade-off can be dependent on specific conditions, as plants thriving in nutrient-rich environments may not necessarily be prioritized to favor one response over the other [181]. Each plant hormone has a recognized, distinct signaling pathway [182]. Signal transduction and transcription regulation include cross-regulation of these pathways [183]. Transcription factors shared between pathways and independent TFs regulating target genes shared by both pathways can cause cross-regulation of transcription. The transcriptional responses to various hormones share a small number of genes [39]. The DELLA and JASMONATE-ZIM DOMAIN (JAZ) proteins and NONEXPRESSER OF PR GENES 1 (*NPR1*) are classic examples of hormone cross-regulation. Each of these examples involves proteins that are mostly controlled by a single hormone and that act on that hormone’s route, but they also have an impact on other hormone signaling pathways [184]. A hormone stimulation causes thousands of genes to change in expression [185]. Gene expression changes dynamically and demonstrates an extensive amount of variance throughout time, showing a range of gene expression patterns [186]. Different TFs dynamically regulate genes at different times in response to stress. Jasmonic acid (JA), ethylene (ET), and abscisic acid (ABA) are the three hormones that control the expression of tens to hundreds of TFs; all hormones probably function similarly [187]. Several TFs may target a single gene, and individual TFs may target hundreds to thousands of genes. This enables dynamic and complex expression patterns but presents a substantial issue in determining which TFs regulate these patterns [184]. The primary JA signaling pathway is significantly regulated by the JAZ repressor JAZ10, which encodes both the dominant negative and active forms of the protein [188].

### 6.3. Coordinated Regulation of Gene Expression during Salt Stress

Saline soil causes decreased seed germination rates and plant growth. One of the primary adverse factors impacting food security and agricultural productivity is soil salinization [189]. Plants have developed complex signaling pathways to adapt to changing environmental conditions, typically consisting of signal transducers, secondary signals, plant hormones, and receptors [190]. Transcription factors (TFs), also observed as trans-acting factors, are significant components of abiotic stress signal-transduction pathways. Plant resistance is a result of transcription factors that bind to certain cis-elements and control the expression of a gene family associated with them. The DNA-binding domain, transcriptional regulatory domain, nuclear localization signal (NLS), and oligomerization site are the four basic components of typical transcription factors. In Table 6, the TFs and their domains associated with salinity stress are summarized. The combined action of these structural domains influences the time, space, and mode of action of the regulatory functions of transcription factors [35]. Many transcription factors act as major regulators to select genes, controlling the determination of cell type, development pattern, and specific pathway control [191]. Plants produce and exchange several signals that activate transcription factors in response to biotic and abiotic stressors, including salt, drought, extremes in temperature, and pathogens. Transcription factors bind with corresponding *cis*-acting elements to switch on RNA polymerase and transcribe complexes, therefore creating the transcription and expression of specific genes. Products eventually begin responding to the signal [192].

## 7. Regulation of Salt-Stress-Related Genes

### 7.1. Molecular Mechanisms Controlling the Expression of Salt-Responsive Genes

**Figure 3:**
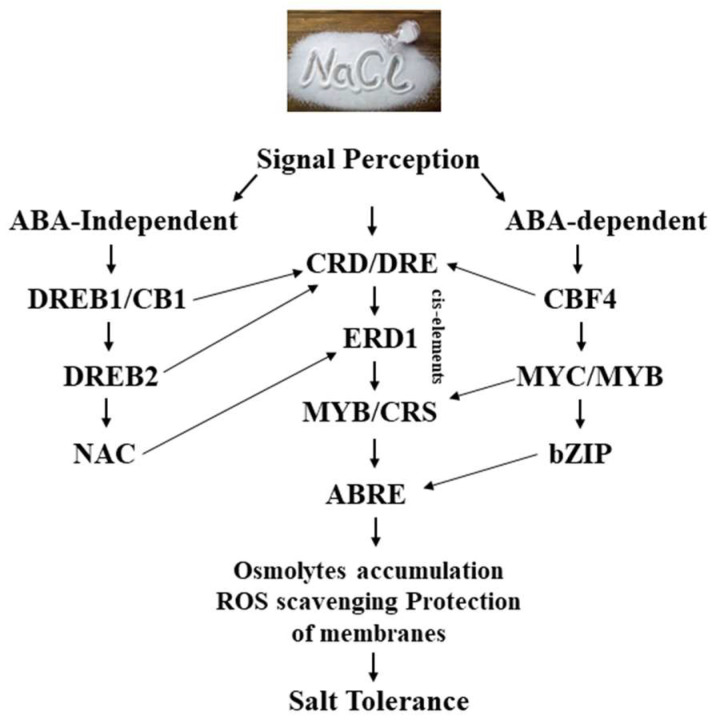
Schematic representation of the salinity stress signal perception and gene expression.

Plants develop a response through the perception and transduction of osmotic and ionic signals, resulting in modifications to cellular characteristics under salt stress. To date, no salinity sensor/receptor information has been reported in plants [226]. On the other hand, the Arabidopsis signaling pathway includes salt excessively sensitive (SOS) and calcineurin B-like (CBL)/CBL-interacting kinases. Higher cytosolic Ca^2+^ concentrations activate the Ca^2+^-dependent protein kinase complex (SOS_2_-SOS_3_), which phosphorylates SOS1 and starts its activity. Furthermore, a novel pathway for the control of vacuolar Na^+^ sequestration modulated by the CBL10-CIPK24 complex was revealed. Furthermore, TFs are dependent on both ABA-dependent and ABA-independent pathways, and salinity stress promotes the increased formation of ABA that triggers signaling (Figure 3). Through phosphorylation, it activates sucrose non-fermenting 1-related protein kinase 2 (*SnRK2*). Plants show inhibition of *snRK2* under control conditions, but in reaction to stress, they bind to PYRABACTIN RESISTANCE 1 (PYR1), a receptor protein. Several downstream TFs are activated or inhibited when *PP2C* is released from *SnRK2* and self-phosphorylates, namely the ABA-responsive factors (ABF) and the abscisic acid (ABA)-responsive element (ABRE)-binding protein (AREB) [227]. The mitogen-activated protein kinases (MAPKs) are another possible option for salt-stress sensing. It is responsible for mediating the homeostasis of secondary stimuli, including oxidative, osmotic, and ionic stresses. It is classified into three distinct classes, determined by the phosphorylation and activation of elements, i.e., MAPK, MAPKK, and MAPKKK.

### 7.2. Epigenetic Modifications and Chromatin Remodeling

Widespread salinity and sodality have adversely impacted large areas of arable land, limiting crop development and lowering land production per person. Understanding the molecular mechanisms underlying each stress and being able to control them to feed the world’s population has become crucial for scientists. In this situation, modifying key histone proteins through methylation, acetylation, and phosphorylation resulted in epigenetic solutions that have been of unknown benefit in affecting plant responses to salt-induced stress [228]. Scientific understanding of the fundamental epigenetic process regulating blooming, germination, fruit ripening, vernalization of fruiting photoperiodism, etc., has been made easier by advances in biotechnology. To assist in the identification of the configurational changes that the genome undergoes during cell differentiation. Studies investigating plant-heritable epigenetic marks have been performed to find out more about natural selection processes and other adaptive responses of plants to their surroundings [229]. Studies on various abiotic stress responses have clearly shown that some abiotic stressors trigger somatic memory through mitotic cell divisions, which can persist for a long period [230]. Memory quickly resets to baseline levels when normalcy can thrive. However, in meiotic cells, some memories influence chromatin. They are heritable and thus have the potential to pass on from parent plants to the stress-free offspring of the generation, which is known as a transgenerational epigenetic inheritance [231]. DNA methylation and histone modifications may have a synergistic effect on stress-induced genes because salinity stress affects the expression of various transcription factors in soybeans [232]. Hypomethylation might be associated with changes in the expression of DNA demethylases under salt stress [233]. When salinity stress was applied, contrasting variations in cytosine methylation patterns were observed in the progenitor and salinity-tolerant wheat cultivar SR3. The responses of contrasting wheat genotypes under NaCl stress could be attributed to the altered expression levels of high-affinity potassium transporters (HKTs) regulated through genetic and epigenetic mechanisms [234]. The hypersensitivity of Arabidopsis thaliana to NaCl stress is attributed to the transcriptional adaptor ADA2b, which is responsible for histone acetyltransferase activity. On the other hand, plant responses are more complicated due to the histone modifications and crosstalk between histone acetylation and cytosine methylation [235]. Both genome-wide DNA methylation and histone modifications are affected by salt stress, and both mechanisms interact to provide a synchronized response to salt stress [236]. Plant responses discussed above under various stress conditions, salinity stress also triggers common reactions including modifications in histones and changes in DNA methylation. The reactions to stress stimuli result in a noticeable shift in chromatin organization and dynamics, facilitating locus-specific gene expression in plants [237]. Researchers observed that the expression of genes, including *SUVH2/5/8*, *ROS1*, *MSH6*, *APUM3*, *MOS6*, and *DRB2* was down-regulated in a few filial generations of saline-stressed Arabidopsis thaliana cultures. The reduction of H3K9ac in the promoter region of the coding sites, the amplification of H3K9me2, and DNA hypermethylation are the causes of this phenomenon [238]. The regulatory agents for the salinity-responsive genes *Glyma11g0200*, *Glyma08g41450*, and *Glyma20g30840* in soybean (*Glycine max*) are increased amounts of H3K4me3, H3K9ac, and reduced levels of H3K9me2 in combination with DNA hypermethylation [232].

### 7.3. Post-Transcriptional and Post-Translational Modifications of Proteins

Gene expression is regulated through two major modes, transcriptional and post-transcriptional regulation mechanisms, for the initiation of transcription, determining whether to activate or repress the process and consequently inflecting the number of proteins synthesized during translation [232]. Plants under stress exhibit a network of regulatory mechanisms involving the reprogramming of the expression of several key genes at both transcriptional and post-transcriptional levels [239]. These regulatory processes are vital for plants to recover and regenerate cellular homeostasis during both the stress period and the subsequent recovery phase. Understanding these regulatory mechanisms of salinity stress response and tolerance at transcriptional and post-transcriptional levels has been considerably clearer by high-throughput sequencing techniques, readily available databases, and in silico tools. Current studies have demonstrated the presence of several significant functions at both the transcriptional and post-transcriptional levels, including regulatory noncoding RNA species, small and micro-RNAs, and others that play crucial regulatory roles in identifying and regulating the effects of salinity stress on important crops [239]. These regulatory elements are considered key roles in enhancing salinity tolerance in crucial crops through genetic engineering. The formation of a Special Issue aims to highlight diverse transcriptional and post-transcriptional regulatory mechanisms involved in sensing, signaling, and responding to salinity stress in significant crops and model plants using a wide variety of biological, analytical, and computational tools. Many researchers have thoroughly explored the physiological, molecular, and biochemical responses to salinity stress. Various strategies, such as melatonin, play a significant role in regulating plant growth, development, and responses to stress [240]. Various genomic and non-genomic strategies are currently undergoing validation to assess their potential to enhance crop salt tolerance. These strategies encompass stress-inducible promoters, protein post-translational modification, halotolerant microbiomes, and the gene resources of halophytes. The development of novel genetic resources for enhancing salt tolerance has been made easier by cutting-edge techniques in plant phenotyping, next-generation sequencing, and molecularly assisted breeding [241]. The draft genome and transcriptome analysis of *Oryza coarctata*, *halophylic rice*, and a wild relative of cultivated rice have been conducted to provide a potential resource for factors related to salinity and submergence stress response [242]. There is a clear varietal difference between indica and japonica rice cultivars related to their varying salinity tolerance capacities, as shown by the transcript profiling of stress-responsive genes and alter in metabolism during salinity [243]. De novo transcriptome assembly, sequencing, and gene expression profiling of *Sosola drummondii*, a salt-stressed halophyte from a saline environment [244]. Chickpea plant response to NaCl stress was examined with DNA polymorphisms identified by whole-genome sequencing and their potential functional implications [245].

## 8. Conclusions

Enhancing NaCl stress tolerance in plants has far-reaching implications that extend beyond agriculture and social aspects. The development and adoption of salt-tolerant crops show a significant increase in more sustainable global food. Research has identified several key TFs concerned with salt-stress response, including members of the AP2/ERF, MYB, and bZIP families. These TFs play crucial roles in regulating the expression of stress-responsive genes. TFs regulate the expression of downstream target genes by binding to specific cis-elements in their promoters. In this review paper, we highlighted the role of TF networks, indicating crosstalk and interaction between plant hormones under salt stress. Further research aims to understand the regulatory mechanisms of transcription factors in plant morphogenesis, therefore facilitating the development of genome-edited plants that hold potential for biotechnological applications.

## Figures and Tables

**Table 1:** WRKY Transcription Factors in Response to Salt Stress.

*WRAKY* Transcriptional Factors			
Plant	Gene	Involved	References
*Fritillaria crassifolia*	*FcWRAKY70*	Salt stress	[40]
*Glycin max*	*GmWARKY17*	Salt stress	[41]
*Zea mays*	*ZmWARKY17*	Salt stress	[42]
*Sorghum bicolor*	*SbWARKY30*	Salt stress	[43]
*Gossypium barbadense*	*GbWARKY1*	Salt stress	[44]
*Ipomoea batatas*	*IbWARKY47*	Salt stress	[45]
*Penicillium glaucum*	*PgWARKY33/62*	Salt stress	[46]
*Sorghum bicolor*	*SbWARKY50*	Salt stress	[47]
*Vitis pseudoreticulata*	*VpWARKY1* and *VpWARKY2*	Salt stress	[48]
*Malus baccata*	*MbWARKY5*	Salt stress	[49]
*Cucurbita pepo*	*CmWARKY*	Salt stress	[50]
*Pyrus betulifolia*	*PbWARKY40*	Salt stress	[51]
*Citrullus lanatus*	*ClWARKY20*	Salt stress	[52]
*Malus xiaojinensis*	*MxWARKY53*	Salt stress	[53]
*Malus xiaojinensis*	*MxWARKY64*	Salt stress	[54]
*Arachis hypogaea* L.	*AhWARKY75*	Salt stress	[55]
*Myrothamnus flabellifolia*	*MfWARKY70*	Salt stress	[56]
*Arabidopsis thaliana*	*AtWRKY33*	Salt stress	[57]
*Gossypiumhirsutum*	*GhWRKY34*	Salt stress	[58]
*Populuseuphratica*	*PeWRKY1*	Salt stress	[59]
*Solanum lycopersicum*	*SlWRKY23*	Salt stress	[60]

**Table 2:** bZIP Transcription Factors in Response to Salt Stress.

*bZIP* Transcriptional Factors			
Plant	Gene	Involved	References
*Arabidopsis thaliana*	*AtbZIP17*	Salt stress	[61]
*Boehmeria nivea*	*BnbZIP2*	Salt stress	[62]
*Capsicum annuum*	*CAbZIP1* and *CabZIP25*	Salt stress	[63,64]
*Glycine max*	*GmbZIP2*	Salt stress	[65]
*Oryza sativa*	*OsbZIP05*	Salt stress	[66]
*Triticum aestivum*	*TabZIP8* and *TabZIP14B*	Salt stress	[67,68]
*Tamarix hispida*	*ThbZIP1* and *TabZIP15*	Salt stress	[69,70]
*Jatropha curcas*	*JcbZIP34, JcbZIP36, JcbZIP49* and *JcbZIP50*	Salt stress	[71]
*Zay mays*	*ZmbZIP4*	Salt stress	[72]
*Solanum lycopersicum*	*SlbZIP1*	Salt stress	[73]
*Solanum tuberosum*	*StbZIP25* and *StbZIP65*	Salt stress	[74,75]
*Setaria italica*	*SibZIP35*	Salt stress	[76]
*Vaccinium* sp.	*VabZIP12*	Salt stress	[77]
*Isatis indigotica*	*IibZIP*	Salt stress	[78]
*Ipomoea batatas*	*IbbZIP1*	Salt stress	[79]

**Table 3:** MYB Transcription Factors in Response to Salt Stress.

*MYB* Transcriptional Factors			
Plant	Gene	Involved	References
*Oryza sativa*	*OsMYB6* and *OsMYB48-1*	Salt stress	[80,81]
*Arabidopsis thaliana*	*AtMYB44* and *AtMYB2*	Salt stress	[82]
*Triticum aestivum* L.	*TaMYB19-B,*	Salt stress	[83]
*Glycine max*	*GmMYB76*	Salt stress	[84]
*Nicotiana tabacum*	*PsnMYB108*	Salt stress	[85]
*Solanum lycopersicum*	*SlMYB102*	Salt stress	[86]
*Leymus chinensis*	*LcMYB2*	Salt stress	[87]
*Arachis hypogaea*	*AhMYB94*	Salt stress	[88]
*Zea mays*	*ZmMYB3R*	Salt stress	[89]
*Medicago sativa*	*MsMYB4*	Salt stress	[90]
*Vitis vinifera*	*VhMYB2*	Salt stress	[91]
*Casuarina equisetifolia*	*CeqMYB4*	Salt stress	[92]
*Gossypium hirsutum*	*GhMYB108*	Salt stress	[93]
*Actinidia chinensis*	*AcMYB3R*	Salt stress	[94]
*Chrysanthemum*	*CmMYB2*	Salt stress	[95]
*Prunus avium*	*PacMYBA*	Salt stress	[96]
*Malus domestica*	*MdoMYB121*	Salt stress	[97]
*Betula platyphylla*	*BplMYB46*	Salt stress	[97]
*Panax ginseng*	*PgMYB*	Salt stress	[98]
*Lilium lancifolium* L.	*LlMYB3*	Salt stress	[99]
*Dendrobium catenatum*	*DcMYB*	Salt stress	[100]

**Table 4:** NAC transcription factors in response to salt stress.

*NAC* Transcriptional Factors			
Plant	Gene	Involved	References
*Ipomoea pes-caprae*	*IpNAC10*	Salt stress	[101]
*Oryza sativa*	*OsNAC5* and *OsNAC6*	Salt stress	[102]
*Actinidia chinensis*	*AvNAC030*	Salt stress	[103]
*Helianthus annuus*	*HaNAC-1*	Salt stress	[104]
*Cicer arietinum* L.	*CarNAC1*	Salt stress	[105]
*Brassica napus*	*BnNAC5*	Salt stress	[106]
*Chrysanthemum grandiflora*	*ClNAC9*	Salt stress	[107]
*Lilium pumilum*	*LpNAC13*	Salt stress	[108]
*Zea mays*	*ZmSNAC1*	Salt stress	[109]
*Pennisetum glaucum*	*PgNACs*	Salt stress	[110]
*Populus*	*PtNAC024* and *PtNAC182*	Salt stress	[111]
*Glycine max*	*GmNAC06*	Salt stress	[112]
*Cucumis melo* L.	*CmNAC14*	Salt stress	[113]
*Musa acuminata*	*MusaNAC042*	Salt stress	[114]
*Cicer arietinum*	*CarNAC4*	Salt stress	[115]
*Malus domestica*	*MdNAC047*	Salt stress	[116]
*Sorghum bicolor* (L.)	SbNAC1	Salt stress	[117]
*Malus baccata*	*MbNAC25*	Salt stress	[118]
*Tamarix hispida*	*ThNAC13*	Salt stress	[119]
*Medicago sativa* L.	*MsNAC058*	Salt stress	[120]
*Vitis vinifera*	*VvNAC17*	Salt stress	[121]
*Avena sativa*	*AsNAC9* and *AsNAC4*	Salt stress	[121]

**Table 5:** The importance of transcription factors in salt-stress signaling and those involved in four TF families responsible for abiotic stress tolerance.

Transcription Factor	Response
WRKY	Reduces harmful ROS levels and ABA signaling positive regulators to protect cell membranes [122] and reduces salt tolerance [41].
NAC	Triggers germination/growth rate, higher seed germination under high salinity, and osmotic stress [123].
bZIP	Scavenges reactive oxygen species by modifying specific gene expression to improve the salt tolerance of the plant [69]. Involved in ABA response [67,124]. Inhibits seed germination and seedling growth [125].
MYB	Response of the transcriptome to osmotic stress and osmolyte formation and maintains root growth under salinity stress [126].

**Table 6:** A summary of the transcription factors associated with salt stress.

Family	DNA-Binding Domains	Cis-Acting Element	Plant Species	Genes Involved in Salt Response	Reference
NAC	NAC domain	NAC recognition sequence (TCNACACGCATGT)	*Arabidopsis thaliana*	*AtNAC2 AtNAC019* *AtNAC055* *AtNAC072*	[193,194]
			*Oryza sativa*	*OsNAC6* *SNAC1* *SNAC2*	[195,196]
			*Cicer arietinum*	*CarNAC5*	[197]
			*Gossypium hirsutum*	*GhNAC4* *GhNAC6*	[198]
			*Triticum aestivum*	*TaNAC4*	[199]
			*Setaria italica*	*SiNAC*	[200]
MYB	MYB domain	MYBR (TAACNA/G)	*Arabidopsis thaliana*	*AtMYB2 AtMYB4* *AtMYB6* *AtMYB7* *AtMYB44* *AtMYB73*	[201,202]
			*Glycine max*	*GmMYB76 GmMYB92*	[84]
WRKY	WRKYGQK domain	W-box (TTGACT/C)	*Oryza sativa* *Nicotiana benthamiana*	*OsWRKY45* *NbWRKY*	[139] [203]
			*Glycine max*	*GmWRKY21 GmWRKY54 GmWRKY13 GmMYB177*	[84,204]
ERF/DREB	AP2/ERF domain	DRE sequence, GCC box (AGCCGCC), and (TACCGACAT)	*Arabidopsis thaliana*	*DREB2A* *DREB2C*	[205,206]
			*Oryza sativa*	*OsDREB1A OsDREB1C OsDREB1F OsDREB2A*	[207,208]
			*Hordeum vulgare*	*HvDRF1* *HvDREB1*	[209,210]
			*Zea mays*	*ZmDREB2A*	[211]
			*Pennisetum glaucum*	*PgDREB2A*	[212]
			*Setaria italica*	*SiDREB2*	[213]
			*Capsicum annum*	*CaDREBLP1*	[214]
			*Artiplex hortensis*	*AhDREB1*	[215]
			*Glycine max*	*GmDREBb GmDREBc GmDREB2*	[216,217]
			*Cicer arietinum*	*CAP2*	[218]
			*Salicornia brachiata*	*SbDREB2A*	[219]
bZIP	bZIP domain	GLM (GTGAGTCAT), ABRE (CCACGTGG), GCN4-like-motif (GTGAGTCAT),	*Arabidopsis thaliana*	*ABF2* *ABF3* *ABF4*	[220]
			*Glycine max*	*GmbZIP44* *GmbZIP62* *GmbZIP78* *GmbZIP132*	[221]
			*Triticum aestivum*	*Wlip19*	[222]
			*Oryza sativa*	*OsABI5* *OsbZIP23*	[223]
			*Zea mays*	*ZmbZIP17*	[224]
			*Solanum lycopersicum*	*SlAREB*	[225]

## Data Availability

Not applicable.
